# Possible contribution of IMRT in postoperative radiochemotherapy for rectal cancer: analysis on 1798 patients by prediction model

**DOI:** 10.18632/oncotarget.10228

**Published:** 2016-06-22

**Authors:** Wen-Yang Liu, Nicola Dinapoli, Xin Wang, Elisa Meldolesi, Maria Antonietta Gambacorta, Giuditta Chiloiro, Hua Ren, Hui Fang, Ning-Ning Lu, Yu Tang, Lei Deng, Jian-Yang Wang, Hao Jing, Qin Xiao, Yan-Ru Feng, Ye-Xiong Li, Shu-Lian Wang, Yong-Wen Song, Yue-Ping Liu, Wei-Hu Wang, Vincenzo Valentini, Jing Jin

**Affiliations:** ^1^ Department of Radiation Oncology, Cancer Hospital, Chinese Academy of Medical Sciences and Peking Union Medical College, Beijing, China; ^2^ Department of Radiation Oncology, Università Cattolica del Sacro Cuore, Rome, Italy; ^3^ Department of Radiation Oncology, Hunan Cancer Hospital, The Affiliated Cancer Hospital of Xiangya School of Medicine, Changsha, China

**Keywords:** rectal cancer, nomogram, survival, postoperative radiochemotherapy, intensity-modulated radiation therapy

## Abstract

The evidence for adjuvant therapy in locally advanced rectal cancer after TME surgery is sparse. The aim of this study was to identify predicting factors of overall survival (OS) in these patients and combine them into a nomogram for individualized treatment. 1798 patients with pathologically staged II/III rectal adenocarcinoma treated by radical TME surgery from a single center's database were reviewed. The nomogram was derived by Cox proportional hazards regression. Its performance was assessed by concordance index and calibration curve in internal validation with bootstrapping. Pooled Cox model analysis identified age, sex, grade of histology, pathological T and N stage, residual tumor, concurrent radiochemotherapy (RTCT), adjuvant chemotherapy cycles (CT), radiotherapy (RT) unexpected interruption days and intensity-modulated radiation therapy (IMRT) as significant covariates for 5-year OS (P<0.05). Postoperative RTCT, CT and IMRT all improved OS. The proposed model can predict 5-year OS with a C-index of 0.7105. IMRT significantly benefited OS in multivariate analysis (p=0.0441).

In conclusion, our nomogram can predict 5-year OS after TME surgery for locally advanced rectal cancer with simple and effective advantage. This model may provide not only baseline OS estimate but also a tool for candidates selecting of adjuvant treatment in prospective studies.

## INTRODUCTION

Rectal cancer is one of the most common cancers in the world. Preoperative concurrent chemoradiotherapy (RTCT) is recommended as the first choice for locally advanced rectal cancer (LARC) based on several randomized controlled trials [1–4], yet in 2010, only 47.3% of such patients received preoperative radiotherapy (RT) in practice based on SEER program [5]. However, although resection based on the principles of total mesorectal excision (TME) has become the cornerstone of multimodality treatment since 1990s [6, 7], the evidence supporting the overall survival (OS) benefit from postoperative treatment is still sparse.

Many published studies have developed nomograms which are successful in predicting oncology events. Recently a study provided a nomogram based on five RCT in preoperative setting which accurately predicted the rate of overall survival, local recurrence and distant metastases, and that was successfully validated by a Chinese dataset [8, 9]. The aim of our study was to identify similar predicting factors of overall survival in postoperative rectal cancer patients and combine them into a nomogram for individualized treatment and further clinical trial design.

## RESULTS

### Follow-up analysis

Patients eligible for the analysis were 1798, with a median follow-up time of 59.8 months, event rates at 5 years of follow-up were 23.8% for OS, and the 5-year OS was 73.8%. The clinical features are shown in Table 1.

**Table 1:** Clinical characteristics of 1798 patients

	no. (%)		no. (%)		no. (%)
Age		Grade of histology		Type of local surgery	
Median	56	High	117 (6.5)	LAR	1230 (68.4)
Range	22-90	Moderate	1378 (76.6)	APR	506 (28.1)
Gender		Low	247 (13.7)	Hartmann	23 (1.3)
Female	751 (41.8)	Unknown	56 (3.1)	LE	26 (1.4)
Male	1047 (58.2)	Pathology T stage		Unknown	13 (0.8)
ECOG		T1 or T2	178 (10.5)	RT	1680 (93.4)
0	549 (30.5)	T3	1543 (85.8)	IMRT	601 (33.4)
1	810 (45.1)	T4	64 (3.6)	RTCT	1414 (78.6)
2	6 (0.3)	Unknown	3 (0.2)	Adjuvant CT cycles	
Unknown	433 (24.1)	Pathology N stage		No	787 (43.8)
Tumor location		0	745 (41.4)	1-6	680 (37.8)
0-5cm	694 (38.6)	1	607 (33.8)	>6	270 (15.0)
5.1-10cm	693 (38.5)	2	425 (23.6)	Unknown	61 (3.4)
10.1-15cm	149 (8.3)	Unknown	21 (1.2)		
Unknown	262 (14.6)				

### Pooled cox model analysis

**Figure 1:**
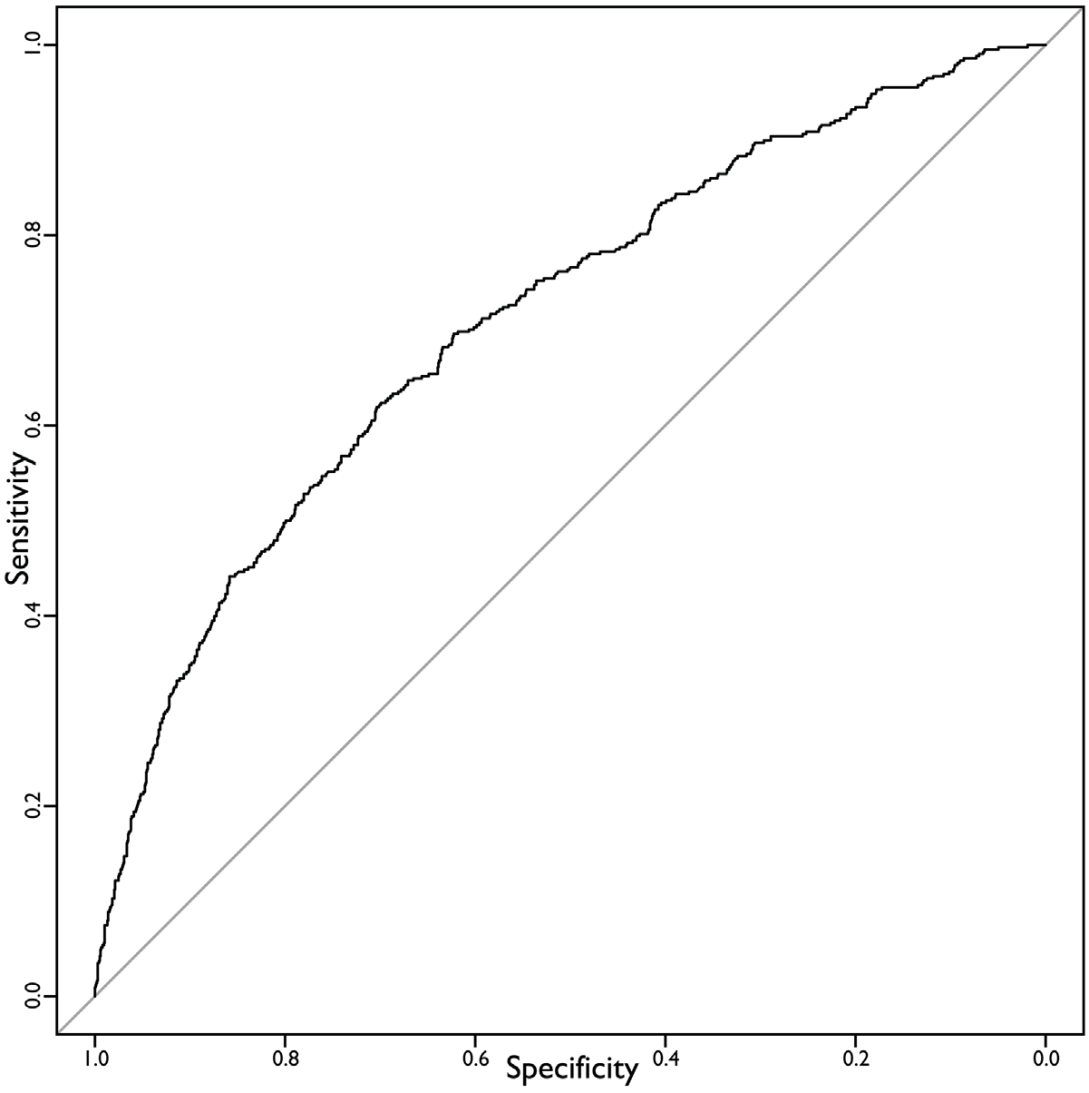
ROC curve of the final predictive model AUC=0.7105.

The survival analysis based on pooled 20 imputed dataset was conducted by multivariate Cox model, the results are in Table 2. Based on the selected significant covariates, the overall P-value of each model calculated for each dataset was always <1^−16^ meaning that the overall regression fit was largely significant. Table 2 also shows that in Kaplan-Meier analyses, baseline characteristics including gender, age and ECOG correlate significantly with OS. Variables of tumor, including pathological T stage, N stage and grade of histology have very significant impact on OS. In this analysis, adjuvant chemotherapy and concurrent radiochemotherapy improve prognosis significantly, even IMRT technique increases OS with marginal significance. AUC analysis was defined for each one of the imputed dataset and corresponding Cox model. The mean value of the AUC was 0.7226 (range 0.7187 - 0.7273). In bootstrap method, the corrected mean of AUC values was 0.7157 (range 0.7108 - 0.7217), not significantly different from the start AUC mean value. The final model was designed by using pooled coefficients derived from the analysis and its final performance in terms of c-index (AUC) was 0.7105. The plot of its ROC curve is shown in Figure 1. Finally the correlation analysis among covariates didn't show significant correlation among them.

**Table 2:** Summary of multivariate Cox analysis and Kaplan Meier univariate analysis

Name	Cox multivariate analysis				Kaplan-meier univariate analysis			
	Parameter estimante	Std error	95% CI	P-value	Sub-class	Pts number	5-Years OS (%)	P-value
Age	.0156	.0046	.0065~.0246	.0008	<50	565	75.9	.003
					51~60	574	73.7	
					61~70	489	74.9	
					>70	170	65.1	
Sex	.2235	.1008	.0258~.4211	.0267	Female	751	76.0	.039
					Male	1047	72.4	
ECOG	.2455	.1261	.0033~.4943	.0531	0	549	79.6	<.0001
					1-2	816	68.1	
Grade of histology	.4582	.1092	.2442~.6723	<.0001	1	117	86.4	<.0001
					2	1378	76.2	
					3	247	57.2	
RTCT	−.2532	.1142	−.4770~-.0292	.0267	No	380	70.7	.015
					Yes	1415	74.9	
pT stage	.4117	.1272	.1624~.6609	.0012	T1	23	90.9	<.0001
					T2	165	78.1	
					T3	1543	73.9	
					T4	64	55.6	
pN stage	.6254	.0683	.4915~.7592	<.0001	N0	745	84.9	<.0001
					N1	607	73.4	
					N2	425	54.6	
Residual tumor	.6316	.0882	.4587~.8044	.0001	R0	1706	75.8	<.0001
					R1	14	52.7	
					R2	59	24.4	
adjuvant CT cycles	−.0534	.0161	−.0849~−.0219	.0009	0~5	1151	72.6	.026
					>5	585	75.9	
RT interruption days	.0169	.0059	.0053~.0285	.0045	0-1	1619	74.6	.044
					>1	90	67.5	
IMRT	−.2508	.1244	−.4949~0.0066	.0441	Yes	590	76.0	.052
					No	1013	72.4	

### Calibration and discrimination

**Figure 2:**
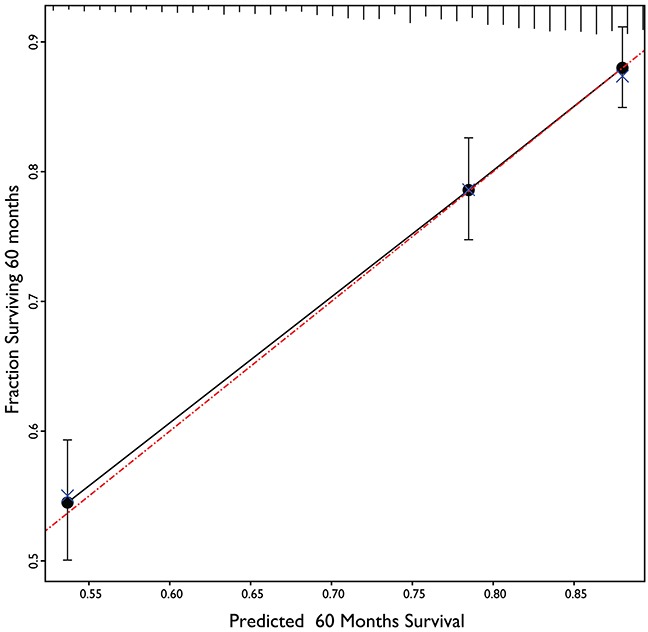
Calibration plot of 5 years overall survival prediction model The calculated values of three prognostic groups (black circles) lie very close to the reference line of perfect calibration (red dash-dot line). The blue X shows the small movements in calibration achieved by bootstrapping procedure to decrease the overfitting of the model.

**Figure 3:**
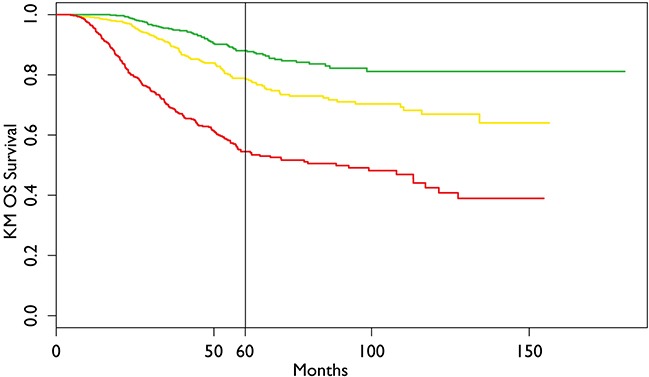
Kaplan Meier Overall Survival of the dataset with three prognostic groups The vertical line refers to the chosen time for model creation (60 months).

The result of calibration of the final model is in Figure 2. It shows almost perfect concordance between the predicted and the actual outcome obtained by Kaplan Meier overall survival estimator. The results of discrimination procedure are set up according to criteria 1b of TRIPOD statement [10]. In order to discriminate among different risk classes three categories of patients have been characterized by ranking them according the value of linear predictor of Cox model. The three survival curves are shown in Figure 3.

### Nomogram

**Figure 4:**
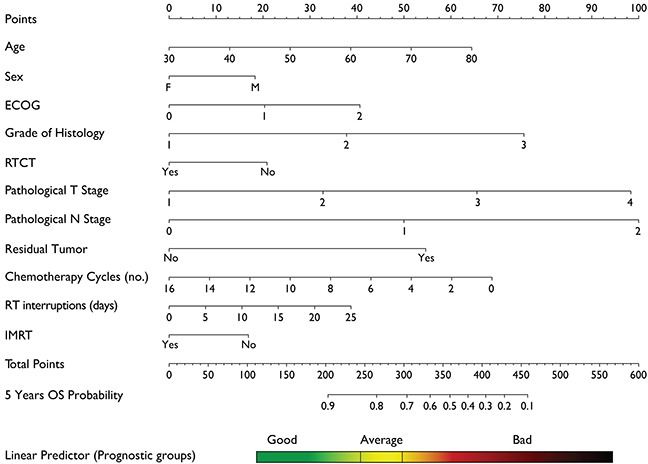
Nomogram for calculation of 5 years Overall Survival Each value in the covariates corresponds to a given score that can be obtained in the top line of the nomogram (“Points”). After summing all the scores for each covariate value the final sum has to be identified in the line “Total Points”. Tracing a vertical line from “Total Points” line down to “5 Years OS Probability” line you can read the expected survival probability assigning the patients to one of the three prognostic groups used for model calibration.

The final nomogram (Figure 4) was generated for calculating the 5 years predicted overall survival. In order to compute the prediction value of a new case, each covariate has to be selected and the corresponding score on the top line has to be summed to other scores. The final value of score sum gives the corresponding value in the predicted survival at 60 months in the bottom line. The positioning of the patient within a prognosis group can be easily be achieved looking at the “linear predictor” reference rectangle, where the color code is the same as color code in Kaplan Meier survival curves in Figure 3.

## DISCUSSION

Based on a large database from our single center, we have developed a 5-year OS prediction model for staged II/III rectal cancer patients who received TME surgery. The relevance of prognostic factors in our model fit well with previous studies [11–14]. Given the distribution of treatment modality of this cohort, the model can support decision making in clinical practice and candidates selecting for future trials in postoperative LARC treatment.

In rectal cancer, prospective trials and retrospective comparisons only indicated the less toxicity and treatment response on par (or at least not inferior) with IMRT to those from historical reports of non-IMRT radiation [15]. In this multiple regression analysis, an amazing but controversial finding was that IMRT technique was associated with a significant improvement in OS. To more thoroughly evaluate this finding, given the possible not uniform application of IMRT, we took into account all the significant factors in Pearson test, including sex, age, ECOG, RTCT, pT, pN, chemotherapy and RT interruption days, residual tumor, results indicated no correlation with other significant covariates. Coupled with the Cox multiple analysis, these data suggest that the observed improvement in OS was significant for patients treated with IMRT compared to non-IMRT. Although it is unlikely to design a RCT trial to compare IMRT with conventional radiation on impact of OS, given these encouraging results based on large data, IMRT could be recommended in routine practice in experienced center in postoperative LARC treatment, despite the borderline significance in univariate log-rank test (p=0.052). A further work on different large dataset could be useful to confirm such conclusion by using a different verification dataset.

Some early trials in non-TME era supported postoperative concurrent radiochemotherapy through benefit in both local control and survival [16–19], which lead to the consensus in US for this modality in stage II/III disease [20]. Nowadays, preoperative radiochemotherapy is recommended as the first choice for these patients worldwide, which improves local control and toxicities with comparable OS [1, 21–23]. Unexpectedly, our data showed that the postoperative concurrent radiochemotherapy improved overall survival after TME surgery. To our knowledge, this was also the only one retrospective analysis based on a large cohort to document the effect of postoperative radiochemotherapy in TME era.

After TME surgery, overall survival of LARC patients primarily depends on the occurrence of distant metastases, however, adjuvant chemotherapy in these patients remains controversial despite several RCT have been performed [3, 24–27]. In our model, more cycles of chemotherapy improved prognosis of patients in overall survival. Recently, a large meta-analysis indicated the benefit of adjuvant chemotherapy in DFS and OS [28]. Furthermore, similar results has been confirmed in ADORE study [29] and the previous nomogram in preoperative setting [8]. Further investigation is needed to define the role of adjuvant chemotherapy in the multimodal treatment of LARC patients to improve the encouraging findings of our results.

Although postoperative RTCT and CT both benefited OS in our data as mentioned above, debate still continues on whether they are both necessary for all LARC after TME surgery [25, 30]. As TME surgery technique alone can decrease the LR rate from 15%~40% to less than 10% [31–33], and preoperative or postoperative adjuvant radiation can further improve local control despite OS benefits are still controversial [1, 22, 34, 35], and then new questions arise which subgroup of patients is the best candidate for adjuvant treatment to improve OS. In the last decade, there is an evolving and progressive expectation for individualized treatment, our nomogram is a decision tool designed to tailor adjuvant treatments in LARC. For potential applying, our model also proposed three prognostic groups (Figure 3), and different treatment strategies could be considered for each category. For example, when a “bad” prognostic patient (male, age 53 years, pT3N2, ECOG=1, grade of histology=3) after R0 resection is compared with a “good” prognostic one (female, age 43 years, pT3N0, ECOG=0, grade of histology=2), if RTCT with IMRT technique and adjuvant CT are applied, the 5-year OS may increase from 33.0% to 62.0% for “bad” and 86.0% to 90.0% for “good” respectively (Figure 4). Considering the potential morbidity [36, 37], cost of RT and CT, this particular “good” prognostic patient might choose to avoid adjuvant treatment given the minimal expected benefit.

Nomogram has been successfully developed for predicting OS in rectal cancer [8]. In addition to estimates of baseline overall survival, our model also provides individualized estimates of potential benefit from adjuvant radiochemotherapy, chemotherapy and IMRT technique. Future prospective studies are needed to more accurately refine indications of adjuvant treatment in these patients. The proposed nomogram performed well in predicting OS with a reliable c-index (0.7105) for internal validation and an excellent result in calibration, but it is still not optimal. Integration with other bioinformation would be expected to increase model accuracy.

There are several limitations that have to be considered for this study. First, the retrospective analysis, despite the strict application of the ontology for decreasing the data collection uncertainty level, did not overcome all uncertainties related to tumor recurrence pattern, surgical quality control by pathologists and treatment heterogeneity (especially chemotherapy regimens). Second, the span of time in this dataset is 10 years, a question might be raised about whether there's change of diagnosis technique through time. Finally, before generalized applying of this nomogram, we expect an independent data to validate using a coherent data ontology classification. In our setting the analysis type 1b according to TRIPOD statement finally ensures the best modeling procedure to take into account the different imputed dataset without splitting the cohort or using different modeling approaches [10].

## MATERIALS AND METHODS

### Study population

In this study a large cohort of patients with histologically proven rectal cancer, pathologically staged as LARC by surgical histological specimen, has been analyzed. The initial dataset included 3995 patients from whom only post-operative radiotherapy cases with complete follow up record were selected. They were treated in our institution since 2000 to 2010. Metastatic patients were excluded from analysis. All patients received a TME procedure, even if the report of the completeness of the mesorectum removal was not available for all patients. Selection criteria included the availability of a follow up time record and the life status of the patients. This study was registered with ClinicalTrials.gov, number NCT02312284.

### The variables

The evaluated variables were classified and collected according to a previously created ontology framework [38]. We included in this analysis the main epidemiological and oncological features for which a percentage of available records not lower than 90% was reachable. Sex, age at the date of diagnosis, tumor location (location was categorized on the basis of the tumor distance measured from the anorectal verge: low, less than 5cm; mid, 5 to 10cm; and high, more than 10cm), grade of histology, concurrent CT (yes/no), type of local surgery (low anterior resection [LAR] and abdominoperineal resection [APR]), pathological T stage, pathological N stage, presence of positive intestinal margin, residual tumor (absence, microscopic or macroscopic), adjuvant RTCT, number of adjuvant chemotherapy cycles, type of postoperative RT field (RT volume included tumor bed for R0 reseciton or Simultaneously Integrated Boost on residual tumor), postoperative RT delivered dose, fraction dose, number of RT unexpected interruption days, and IMRT technique were the analyzed features. Overall survival was selected as evaluated outcome, all causes of death at 5-year were included.

### Statistical analysis

Overall missing data was 9.3% among this patients subset, allowing to perform the imputation procedure with a reasonable safety [39]. In order to ensure that the maximum number of patients was valuable without excluding patients missing only few records, a Multivariate Imputation by Chained Equations procedure was adopted [40]. This procedure allows to achieve more robust models by pooling the results calculated from each imputed datasets analysis, without decreasing the overall informative power due to patients exclusion. This imputation process takes into account for the process that created the missing data, preserves the relations in the data, and preserves the uncertainty about these relations. Imputation process multivariate analysis, by Cox proportional hazards regression, was performed using 20 imputed dataset derived from original one. The inclusion of covariates was achieved by backwards elimination of not significant ones deleting those showing P-value>0.10 and considering significant covariates with P-value ≤0.05. Possible correlation among different covariates in the final model was evaluated by using Pearson correlation test. In order to analyze the performance of the models analysis of the Area Under the Curve (AUC) of Receiving Operating Characteristic (ROC) and calibration on Kaplan-Meier predictors were performed. Both AUC and calibration procedures were tuned using bootstrap in order to decrease the overfitting in the original models. The final results of coefficients, p-values in Cox models, AUC and calibration plots were pooled by calculating means of single imputed dataset.
